# Threat discrimination of real-world social interactions in schizotypal traits

**DOI:** 10.3758/s13423-025-02821-3

**Published:** 2026-02-17

**Authors:** Akila Kadambi, Sophia Baia, Elinor Yeo, Hongjing Lu

**Affiliations:** 1 Department of Psychology, UCLA, Los Angeles, CA USA; 2 Department of Psychiatry and Biobehavioral Sciences, David Geffen School of Medicine, UCLA, Los Angeles, CA USA; 3 Department of Statistics, UCLA, Los Angeles, CA USA

**Keywords:** Social cognition, Threat Processing, Schizotypy, Visual perception

## Abstract

Threat detection is compromised across the schizophrenia spectrum, often revealed by paranoia and delusions. Threat difficulties extend to nonclinical populations with liability toward schizophrenia. A key source of these difficulties may be due to hyper-sensitivity to social stressors in real-world environments. In a large, nonclinical sample (*N* = 161), we measured the influence of social context to threat detection in social interactions. Social interactions were captured in naturalistic videos and validated as threatening or nonthreatening. Deep learning models were employed to re-render the videos by parsing different amounts of social context depicted in these interactions. Then, we measured how threat detection was influenced by individual variability in schizotypal and autistic traits as a function of social context. Individuals with high schizotypal traits showed reduced threat discrimination ability in the presence of more social context, but better threat detection when the interactions were primarily reduced to body kinematics. The effect was more pronounced in individuals higher on suspicious tendencies and odd belief traits in schizotypy, and social communication traits in the autism spectrum. These results suggest that disruptions from social context may underlie threat detection difficulties across the schizophrenia spectrum.

## Introduction

Detecting threat in the environment is a requisite of evolution and is critical for human survival. Threat detection requires accurately matching perceived with real threats based on social and environmental cues. When discrepancies arise between perceived and veridical threat—such as a benign situation misinterpreted as dangerous—this mismatch can often produce maladaptive behavioral responses, as observed in psychopathologies including the schizophrenia spectrum (Freeman et al., 2002).

In the schizophrenia spectrum, threat disturbances are often revealed in paranoia and delusions (Freeman et al., 2002; Henry et al., 2010), significantly impairing life quality and well-being (American Psychiatric Association, 2013). Notably, these difficulties seem exacerbated in situations of social threat, such as when viewing threatening or angry faces (Adolphs, 2008; Bediou et al., 2005; Henry et al., 2010). For instance, schizophrenic patients are worse at identifying (Pinkham et al., 2014) and responding (Varcin et al., 2019) to emotional and valenced faces, as compared with neurotypicals. However, when social influences of the stimuli are accounted for (e.g., by including nonfacial, threatening stimuli, such as snakes), schizophrenics no longer show degraded performance toward threatening stimuli. These findings suggest that threat difficulties in the schizophrenia spectrum could instead arise from the influence of social information (Pinkham et al., 2014; Varcin et al., 2019) that may be due to factors associated with the social valence themselves, such as vigilance-avoidance (a bias away from social stimuli; Green et al., 2003), or reduced attention toward socially evocative neutral stimuli in the environment (e.g., Lipskaya-Velikovsky et al., 2015). Reducing the degree of social context in the negative stimulus might therefore be a useful approach to improve threat detection in the conditions (Bentall et al., 1995; Veling et al., 2016).

Traditional psychological tasks aim to measure threat detection using simplified measurements, such as differences between threatening and nonthreatening stimuli in gaze time, ratings, or affect recognition (Henry et al., 2010). These tasks rely on simplistic or static stimuli, such as in the dot-probe task (Arguedas et al., 2006; Posner, 1980), visually sparse biological motion (Henry et al., 2010; van Boxtel & Lu, 2011, 2012), isolated facial expressions (Green et al., 2003), or using nonsocial but threatening stimuli, such as snakes (Pinkham et al., 2014; see snake-in-the grass task, Öhman et al., 2001). In daily life, however, humans engage in rich, dynamic contexts that change across time and space. Since social threat often involves interpersonal exchanges across different physical interactions and environments, novel measures should be incorporated into the research design that capture realistic social and dynamic properties of daily life.

Experimental paradigms using naturalistic stimuli are gaining increasing popularity in studying the influence of social context and behavioral dynamics (Mobbs et al., 2021). In the past, studying threatening content in naturalistic videos proved challenging due to the complex content of the interactions and the level of emotions involved in videos. Moreover, naturalistic paradigms required technical advances incorporated into the research design to provide a degree of experimental control. Recent advances in deep learning provide ways to overcome these limitations. These models process images and tease apart objects, actors from contextual background through semantic segmentation, minimize emotion related contents by eliminating detailed appearance information in the visual inputs, and eliminate graphic contextual information. These models also present an advantage over other naturalistic methods, such as virtual reality (VR), since they avoid common side effects (dizziness, nausea), while maintaining experimental control by preserving a consistent and realistic view of the entire scene.

Despite extensive evidence for threat-related difficulties in the conditions (Freeman, 2007; Green & Phillips, 2004; Henry et al., 2010; Scholten et al., 2006), no study to our knowledge has directly examined the influence of realistic social context on threat detection in the schizophrenia spectrum. In closely related qualitative work, Fornells-Ambrojo et al. (2008) compared levels of paranoia in patients with persecutory delusions diagnosed with a psychotic disorder relative to a nonclinical group in realistic, socially neutral environments (London underground train) during VR. The patients reported similar levels of paranoia to control participants, suggesting that socially neutral contexts may not necessarily trigger excessive paranoia in patients with delusions, consistent with prior work (Fornells-Ambrojo et al., 2008). However, patients were still significantly more likely to use their own affective state as evidence for the persecutory intent of others. Veling et al. (2016) further examined the relationship between paranoia and social context in VR on patients diagnosed with a psychotic disorder, ultra-high-risk patients for psychosis, siblings of patients with a psychotic disorder, and controls. They observed a direct relationship between paranoid symptoms in patients diagnosed with a psychotic disorder and the degree of social stress present in the environment. While paranoia in all groups increased with environmental social stress, the effect was most pronounced in the patient sample and parametrically degraded by psychosis liability. These studies underscore the influence of social context on paranoid symptoms in psychosis, which is closely linked to schizophrenia (though schizophrenia also involves negative symptoms And tends to be more chronic And Lifelong; American Psychiatric Association, 2013). However, since threat detection was not directly measured nor quantified and the social scene was presented in an artificially rendered environment, it remains unknown whether real-world environments that depict more social context would alter threat detection ability, and/or similarly relate to paranoid symptoms across the schizophrenia spectrum.

In a large, nonclinical sample of participants (*N* = 161), we used recent developments in deep learning to measure the influence of social context on threat detection as a function of psychopathological variability. We chose a nonclinical sample for the following reasons. First, this study is a preliminary investigation of the influence of social context on threat detection from human interactions. Hence, how social context influences threat detection in human interactions remains untested to date, even in nonpatient samples. Second, in using a nonclinical sample, we can avoid confounds associated with medication use, symptom expression variability, and group-level averaging that could mask natural psychopathological variance. Finally, the high prevalence of schizotypal and autism-spectrum traits in the general population (recent estimates range from ~ 1%–3% for a high degree of autistic traits; Ruzich et al., 2015, and 2–10% for schizotypal traits; Fonseca-Pedrero et al., 2018; Raine, 1991) allowed us to explore subprofiles of schizotypy and autism that could strongly relate to differences in detection abilities. Autism-spectrum traits were also critical to include due to known etiological and phenotypic overlap between autism and schizotypy (Couture et al., 2010; cf. Hudson et al., 2023) evident early in development (Barneveld et al., 2011), with similar genetic (Carroll & Owen, 2009) and environmental (Kerns et al., 2015) risk factors, as well as showing strong correlations in the general population (Hurst et al., 2007). Notably, the autism social communication subtype primarily relates to negative schizotypal traits (e.g., flattened affect, social withdrawal; Dinsdale et al., 2013; Zhou et al., 2019). While the exact symptom-dimension overlap remains unclear (Hurst et al., 2007; cf. Crespi & Badcock, 2008), measuring variability in positive schizotypal traits may be useful for differentiating autism from schizophrenia spectrum in the general population, since the condition overlap primarily presents itself in the negative symptoms (Dinsdale et al., 2013). In addition, difficulties with threat detection are also well-documented in the research literature on autism spectrum. Toddlers diagnosed with autism spectrum show reduced attention, yet greater distress to social threat (Macari et al., 2021). Autistic adults and high autistic-trait individuals often exhibit less accurate or delayed responses to threat cues—such as reduced sensitivity to threatening faces (Ashwin et al., 2006; Lassalle & Itier, 2015), slower disengagement from fearful stimuli (Zhang et al., 2024), and reduced alerting to threat (English et al., 2017). Some studies suggest intact but atypical threat prioritization, including a comparable “anger superiority effect” in autistic populations (Isomura et al., 2014; Rosset et al., 2011).

To generate naturalistic interactions, we compiled a range of real-world videos depicting both threatening and nonthreatening interactions across a range of social contexts. These interactions spanned different people and places, including interactions with police officers, security guards, acquaintances, and in different locations. Each threatening and nonthreatening interaction was equally matched according to the setting in which the interaction occurred. The interactions were validated as threatening or nonthreatening on a pilot sample, rank-ordered based on their subjective degree of threat, and later processed through models that produced two types of displays that varied by degree of social context. A new set of participants viewed these interactions parsed by one of these display types and rated the degree of threat in a between-subjects design. Here, we asked the following questions: (1) How does social context impact threat discrimination? (2) Does threat discrimination ability and social context depend on schizotypal and/or autistic traits in the general population?

## Methods

### Participants

A total of 162 participants were recruited from the University of California, Los Angeles (UCLA) Psychology Subject Pool. One participant in the main task was excluded due to scoring more than 4 standard deviations away from the mean on threat discrimination (computed based on the difference between ratings of threatening and nonthreatening videos) for a total of 161 participants included in the analyses (*M*_age_ = 19.96 years, *SD*_age_ = 4.02, men = 38, women = 123). Sample size was based on other large-scale individual differences studies examining the relation between autistic and schizotypal traits and human movements (e.g., Blain et al., 2017; Hudson et al., 2023; Kadambi et al., 2024). The study was approved by the UCLA Institutional Review board. All participants were naïve to the purpose of the study. All participants were provided course credit for their participation after completion of the study. Participants had normal or corrected-to-normal vision.

### Stimuli and apparatus

The experiment was conducted in a dark, quiet space. Stimuli were depicted on a computer monitor 53.1° in width and 40.7° in height. Participants were seated 76.2 cm away from the monitor. Thirty naturalistic interactions were first selected from YouTube and categorized into threatening (*N*_videos_ = 15) or nonthreatening (*N*_videos_ = 15) interactions determined by the first author and three research assistants independently. Threatening videos were selected based on using search descriptions on YouTube which included the following terms or their variants: “threat,” or “threatening interactions,” or “fight.” Nonthreatening videos were constructed to closely match the threatening videos based on location, race, gender, number (between two and three main actors) and age of the actors. If there was a perceivable nonthreatening segment in the long video of threatening events, that segment was used as the corresponding nonthreatening video to match for low-level differences. The nonthreatening segments were selected to avoid any threatening or preparation to commit a threatening interaction. If a threatening video did not have a nonthreatening segment, the video was best matched to a different nonthreatening video by the same criteria. All videos spanned a range of social interactions and demographics, in line with our previous work (Kadambi et al., 2020).

A pilot sample (*N* = 17) independently validated the videos as threatening or nonthreatening. If participants categorized the video as threatening, they were asked to type a short description explaining their decision. The videos were only described by participants if they categorized the video as threatening, for the following reasons. First, we aimed to prompt participants to focus on salient attributes related to threat in their descriptions. Including nonthreatening descriptions could introduce semantic variability that may obscure patterns specific to the construct of interest (i.e., threat). Second, descriptions for videos that participants classified as “nonthreatening” are likely to introduce negation statements, such as “there is no fight” or “the action is not violent.” Semantic analysis at the word level has difficulty in capturing such negation. These validated videos were then separately processed into a patch and body display. Four of the 30 videos were excluded due to technical difficulties in parsing the actions with the RefineNet deep learning model, for a total of 26 videos processed and included in the main experiment. Final video lengths ranged from 8.48 to 31.20 s (*M* = 16.90, *SD* = 6.55) and the mean duration for the threatening videos (18.30 s) was approximately matched to the mean duration for nonthreatening videos (15.60 s). Videos were presented at 600 × 400 pixels. No sound was played during video observation.

**Fig. 1:**
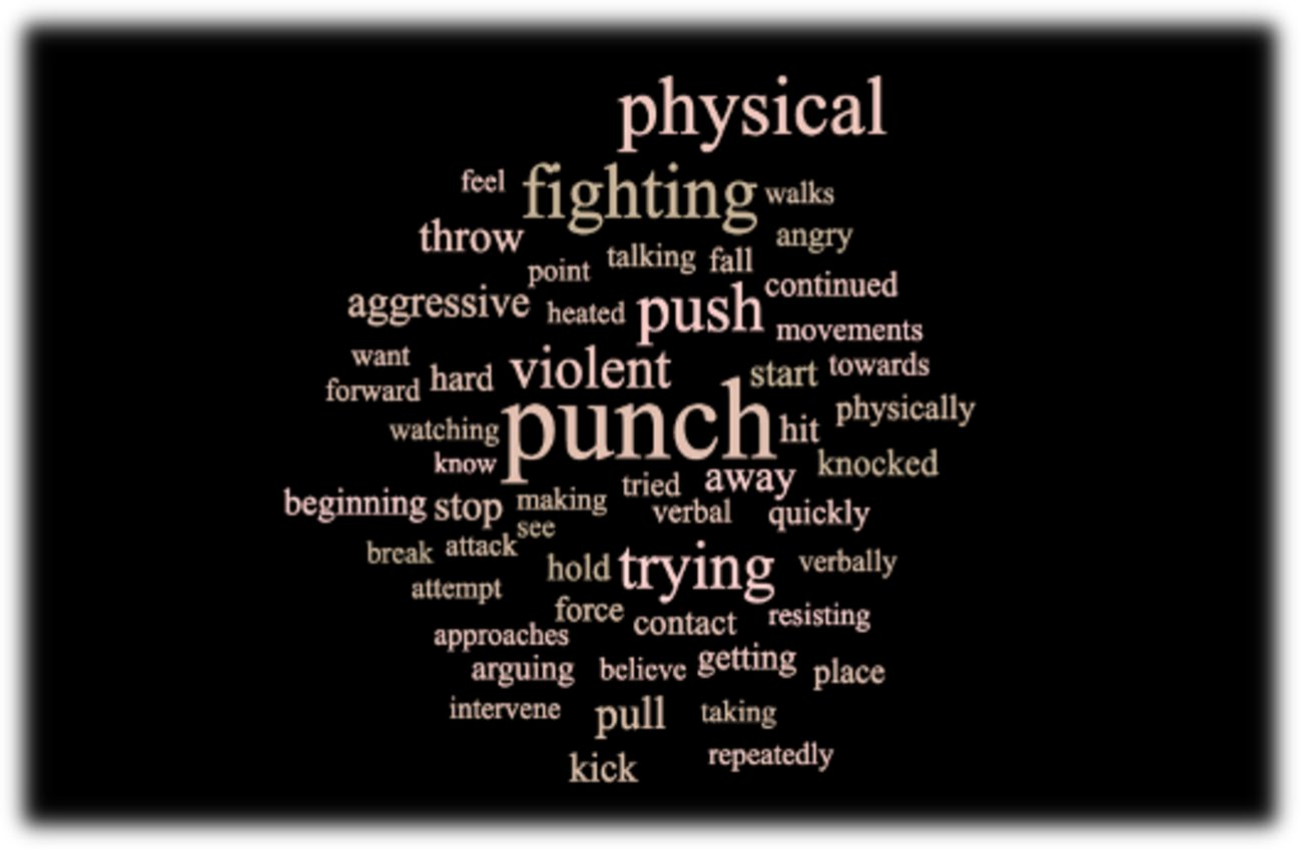
Word cloud depicting word frequencies from participant descriptions for videos classified as “threatening.” Larger sized words indicate more frequent usage. Note that motor words were more frequently used to describe threat in the social interactions

**Fig. 2:**
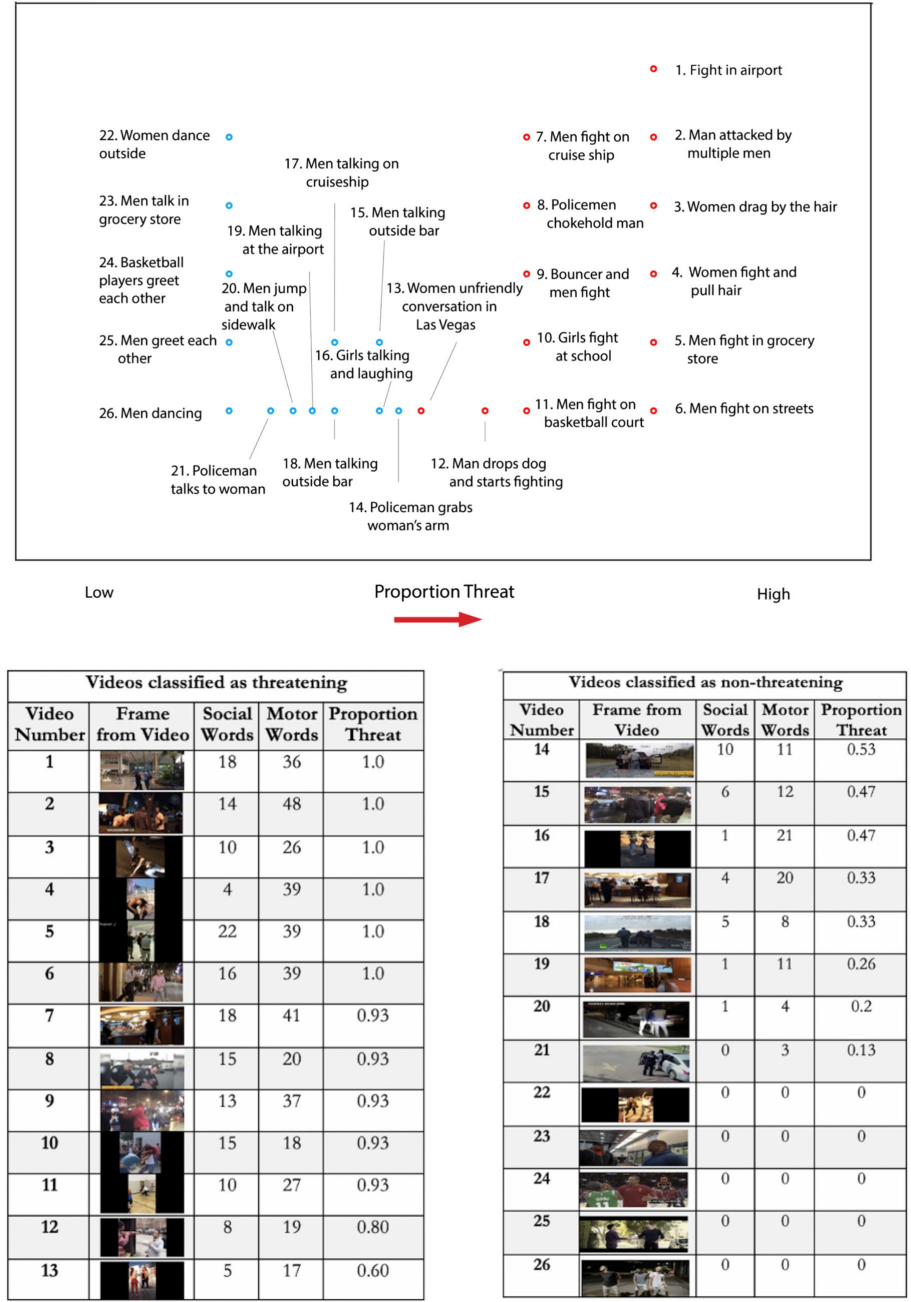
Threat ratings for each video. *Top:* spatial visualization of proportion of threat rankings (only based on the horizontal dimension) for each numbered video. Each video is labeled with a brief description of the interaction for visualization purposes in this figure. Circle color indicates classification of video type: threatening (red) or nonthreatening (blue). *Bottom:* Table of final rank-ordered based on their proportion of threat classification, from most threatening (Video 1) to least threatening (Video 26). Videos were only described by the participant if they judged the video to be threatening. Resultantly, this produced more motor and social words described for threatening than nonthreatening videos

We used Textalyser (https://edutechwiki.unige.ch/en/Textalyser), a program that calculates word frequencies in text (visualized in Fig. 1), to analyze the brief descriptions written for the threatening videos. This allowed us to explore the semantic knowledge used by participants to make their judgments of threat. We then calculated the overall proportion of participants classifying a video as threatening (Fig. 2).

#### Context on threat discrimination as a function of schizotypal and autistic traits

Social context was quantified based on the presence of the following criteria: (1) environmental scene (e.g., background, other individuals, and objects in the scene), (2) demographic characteristics of the main actors (skin color, clothing, age), (3) body morphology. The videos were re-rendered to depict either a blurred visual scene (*patch* display; most context) or color-parsed body limbs against a black background (*body* display; least context).

#### Patch display

**Fig. 3:**
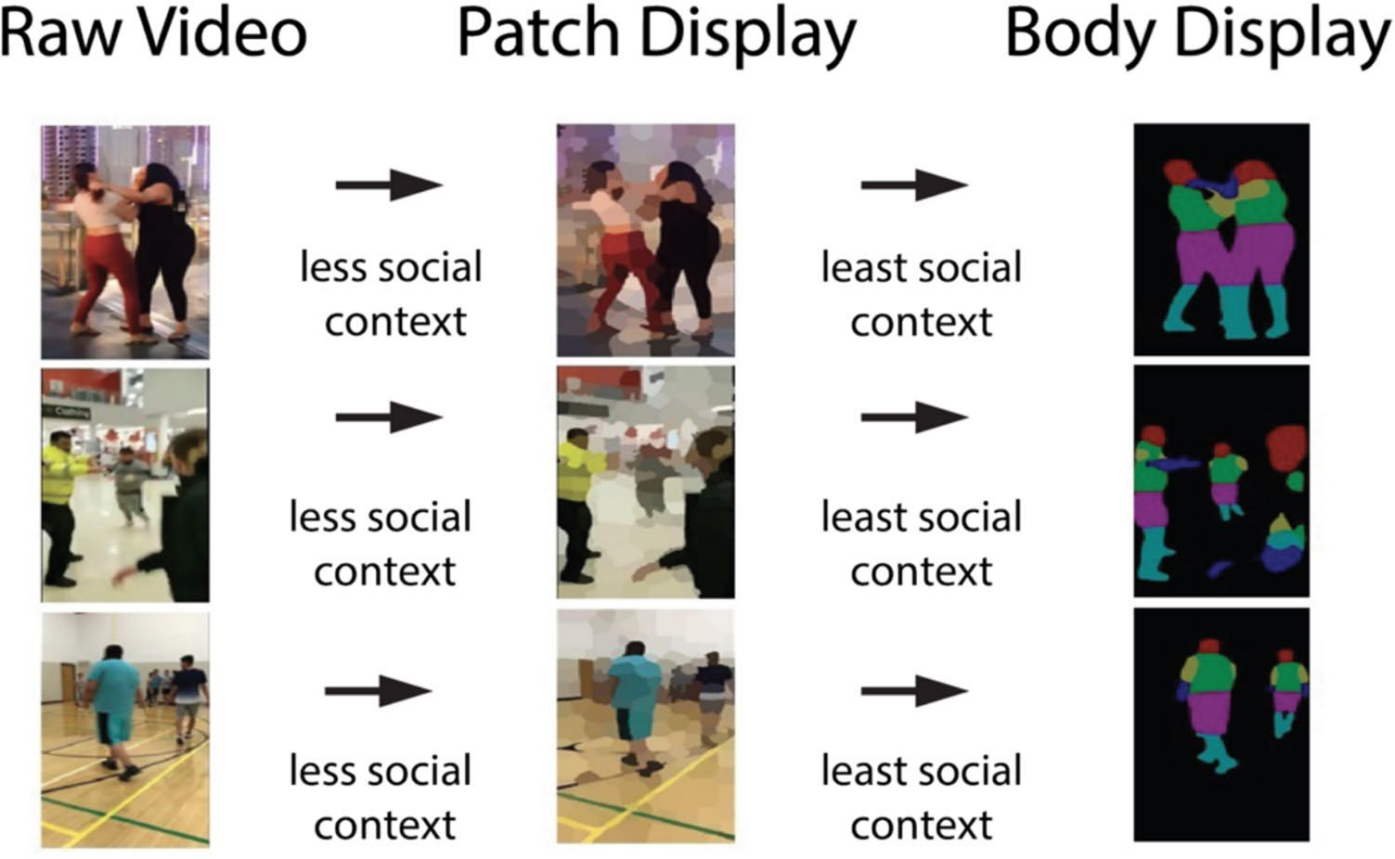
Video parsing from the raw video. The raw video (left column) was converted to the patch display (middle column) that blurred the visual scene and preserved the most social context. The rightmost column depicts video parsing for the body display, in which the interactions were mainly reduced to dynamic body motion. The body display had the least amount of social context. (Color figure online)

To generate the patch display (second column of Fig. 3), each video was parsed through the SuperPixel grouping algorithm in MATLAB (Achanta et al., 2012), which uses local pixel information to group similar pixels based on their color, brightness, and texture into “patches.” This display provided more social context than the body display (described in detail below), as it closely resembled the raw video, but with a blurred effect applied to the entire scene. Despite the blurring, the entire global context of the scene– such as the background environment, individuals, and objects was preserved, along with veridical color information. This controlled blurring was intended to minimize the influence of facial expressions on threat judgments related to schizotypal traits (e.g., Green & Phillips, 2004), without removing key social or environmental cues.

#### Body display

We used the open-source deep learning model, RefineNet, to create the Body Display (third column of Fig. 3). RefineNet uses a multi-layered semantic segmentation algorithm to parse objects or people embedded in visual scenes*.* For each video frame, pixels were segmented by the model to correspond to the following body regions: head, torso, upper arm, lower arm, upper leg, and lower leg (Lin et al., 2017), with each limb displayed in different colors (third column of Fig. 2). Pixels not semantically parsed by the model as a “body” region, were thus depicted in black, resulting in a black visual background. This display had significantly less social context than the patch display, as it preserved body morphology, but eliminated demographic characteristics of the main actors and the background scene. Note that occasionally RefineNet picked up the background actors, but for the most part, only the body parts of the main actors were shown.

### Procedure

In a between-subjects design, participants were randomly assigned a display type (*patch*: more social context; or *body:* less social context) to sequentially view a randomized order of the threatening and nonthreatening videos. After viewing each video, participants rated the degree of threat in the video by selecting a number from 1 (*least threatening*) to 6 (*most threatening*) on the computer. After completion of the task, participants completed the Schizotypal Personality Questionnaire (SPQ; Raine, 1991) and Autism-Spectrum Quotient (AQ; Baron-Cohen et al., 2001). Questionnaire order was counterbalanced between participants.

### Individual difference measures

#### Schizotypal Personality Questionnaire (SPQ)

SPQ (Raine, 1991), is a 74-item self-report questionnaire that measures susceptibility to schizotypy in the general population. The SPQ was developed closely following *DSM-III-R* criteria for schizotypy including three main dimensions of schizotypy: positive, negative, and disorganized (Raine, 1991). SPQ includes nine subscale measures for nine schizotypal features: *ideas of reference*, *excessive social anxiety*, *odd beliefs or magical thinking*, *unusual perceptual experiences*, *odd or eccentric behavior*, *no close friends*, *odd speech*, *constricted affect*, and *suspiciousness*. Although SPQ was developed according to the *DSM-III-R*, it closely matches *DSM-V* criteria for schizotypal personality disorder and so remains applicable (Barron et al., 2014). We utilized the 74-item full-scale SPQ rather than the shorter 32-item SPQ-brief (Cohen et al., 2010) as previous research indicates that the original full-scale SPQ more clearly differentiates the nine subscales (Ford & Crewther, 2014). Responses to each item follow a “yes/no” format with each “yes” response worth 1 point (e.g., “I often feel that others have it in for me”).

#### Autism-spectrum quotient (AQ)

AQ (Baron-Cohen et al., 2001) is a self-report questionnaire measuring the number of autistic traits on a continuum in the general population. The AQ consists of 50 items divided into five categories (containing 10 items each) according to five major dimensions in the autism spectrum: *imagination*, *social skills*, *communication*, *attention to detail*, and *attention switching*. Respondents answer each item on a four-point scale: “definitely disagree,” “slightly disagree,” “slightly agree,” and “definitely agree.” Autism-endorsing answers are worth 1 point each (e.g., “I prefer to do things the same way over and over again”). A cutoff score of 32 points, out of a possible 50 points, indicates the upper threshold of autistic traits in the general population based on 80% of the previously diagnosed autism-spectrum participants and only 2% of the undiagnosed control participants. Although scores may indicate possible clinical levels of Autism, it is not a diagnostic measurement tool.

## Results

All analyses were implemented using SPSS 28.0.0 (IBM SPSS, Armonk, NY), R (Version 4.2.1), and MATLAB R2022A. To measure influences of social context (conveyed by the display type) and video category on threat ratings, we conducted a mixed analysis of variance (ANOVA), specifying one within-subjects factor, video categorization (threat vs. nonthreat videos) and one between-subjects factor, display type (patch vs. body). The within-subjects factor, video categorization, was determined by the proportion of threatening classifications to classify each video as threatening (proportion threat classification > 50%) or nonthreatening (proportion threat classification < 50%) in the pilot study based on responses from different group of participants when viewing the raw videos. In the current study, mean threat rating (1–6; least to most threatening) for each video was used as the dependent measure. Threat detection was operationalized as the extent to which participants subjectively rated threatening videos as more threatening than nonthreatening ones.

**Fig. 4:**
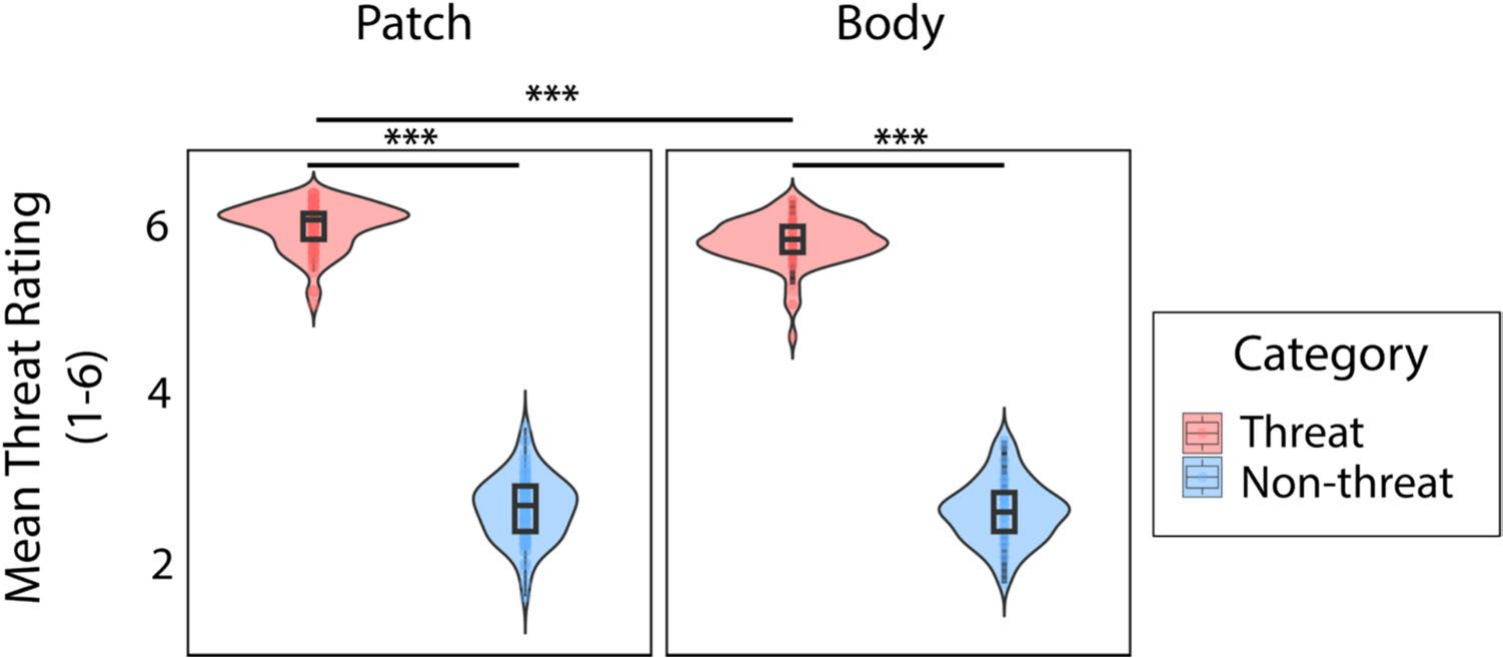
Threat discrimination as a function of display type (patch vs. body). Violin plots show a significant interaction between display type and threat category. Each violin plot includes the kernel density estimation. The boxplot denotes the interquartile range with the median marked by a line. Individual data points are jittered for clarity, with colors corresponding to each display type: red for patch and blue for body. (Color figure online)

Shown in Fig. 4, participants could discriminate threat regardless of the amount of social context in the display, revealed by the main effect of video category, *F*(1,159) = 12,554.67, *p* <.001, η_p_^2^ =.987. The significant two-way interaction effect between display type and video category *F*(1,159) = 6.82, *p* =.010, η_p_^2^ =.041, indicated greater threat discrimination in the patch display than the body display (*p* <.001). Specifically, threatening videos were rated more threatening in the presence of more social context (patch display; *M*_rating_ = 5.54) as compared with less (body display; *M*_rating_ = 5.35). For nonthreatening videos, no difference in threat rating was found between patch and body displays (*p* =.549). Using the mean differences in threat rating between threatening and nonthreatening videos, we also observed that threat was discriminated significantly better in the patch display (*M*_diff_ = 3.34, *SD*_diff_ =.389) compared with the body display (*M*_diff_ = 3.19, *SD*_diff_ =.351), *t*(159) = 2.608, *p* =.010, 95% CI [.0369,.2679]. Thus, the additional social context provided by the patch display facilitated threat discrimination.

### Individual differences analyses

Next, we measured whether the relationship between threat discrimination and individual differences in schizotypy and autism spectrum was influenced by social context in the display type using moderated regression analyses. Dummy-coded display type (patch vs. body) was set as the moderator, with each of the individual difference measures entered as separate predictors. Schizotypal traits were obtained from the SPQ, and autistic-spectrum traits from the AQ. Higher scores on both measures indicate more traits of the respective conditions. Four participants did not complete the SPQ, and one participant (out of the four) also did not complete the AQ. Hence, listwise deletion resulted in 157 participants included in the individual differences analysis. Threat discrimination score, computed as the difference in rating between threatening–nonthreatening videos, was set as the dependent variable. To assess model significance, we computed the significance of the Δ*R*^2^ after adding the interaction term. We checked for outliers using Cook’s distance (observations < 1). Multicollinearity and normality were satisfied using VIF and visual inspection of *Q-Q* plots, respectively. All individual differences were mean centered.

**Fig. 5:**
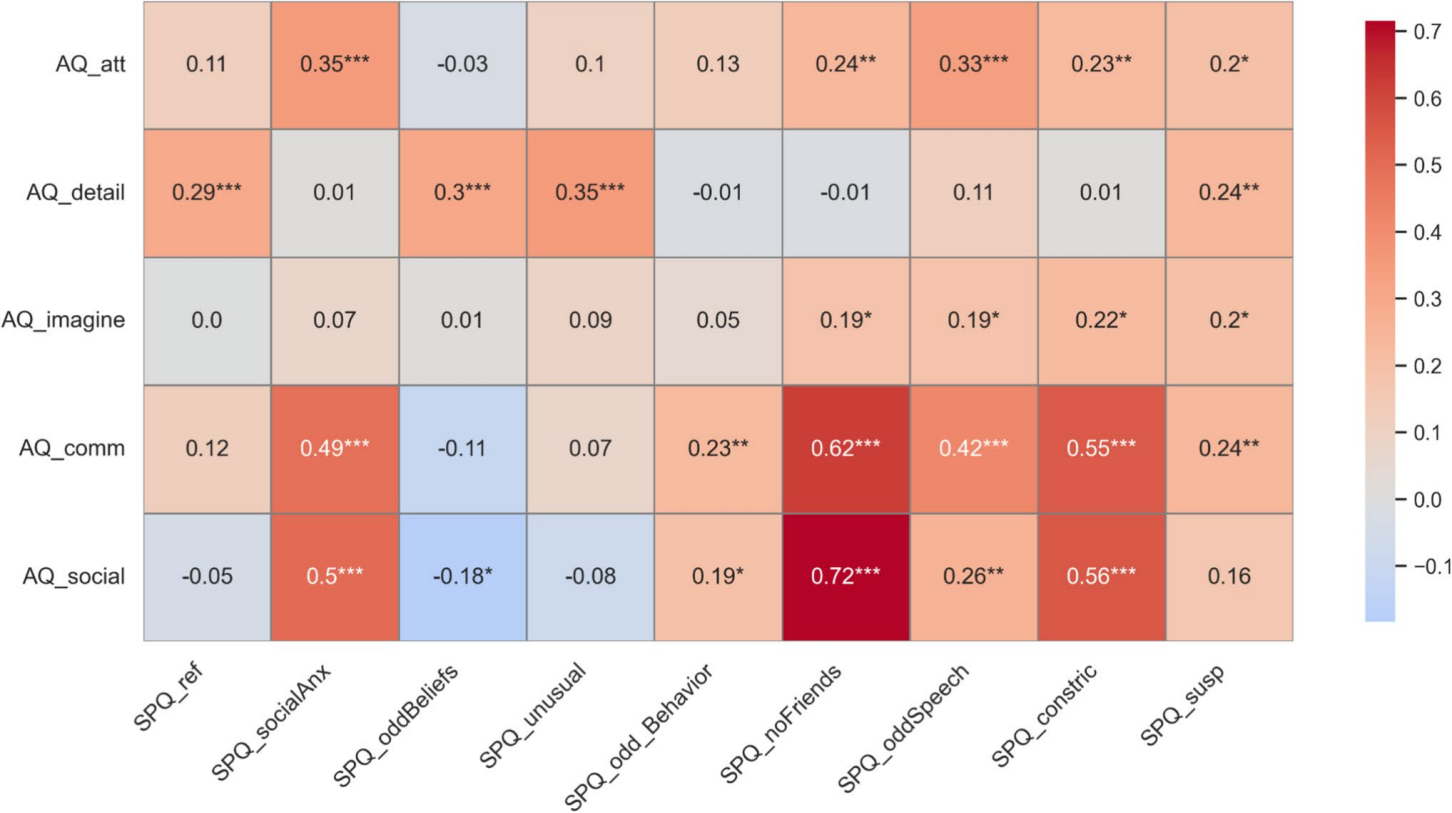
FDR-corrected Spearman correlation plot between Autism Quotient (AQ) and Schizotypal Personality Questionnaire (SPQ) subscales. *Abbrev:* Autism-Spectrum Quotient [AQ]. AQ (-comm): social communication subtype; AQ (-detail): attention to detail subtype; AQ (-social): social skills subtype; AQ (-att): attention switching subtype; AQ (-imagine): imagination subtype; Schizotypal Personality Quotient [SPQ]. SPQ (-ref): ideas of reference subtype; SPQ (-oddSpeech): odd speech subtype; SPQ (-oddBeliefs): odd beliefs subtype; SPQ (-noFriends): no friends subtype; SPQ (-constrict): constricted affect subtype; SPQ (-socialAnx): social anxiety subtype; SPQ (-susp): suspiciousness subtype; SPQ (-oddBehavior): odd behavior subtype. Composite measures are denoted in brackets and bolded. All correlations are FDR corrected: *p <.05, **p <.01, ***p <.001. (Color figure online)

**Fig. 6:**
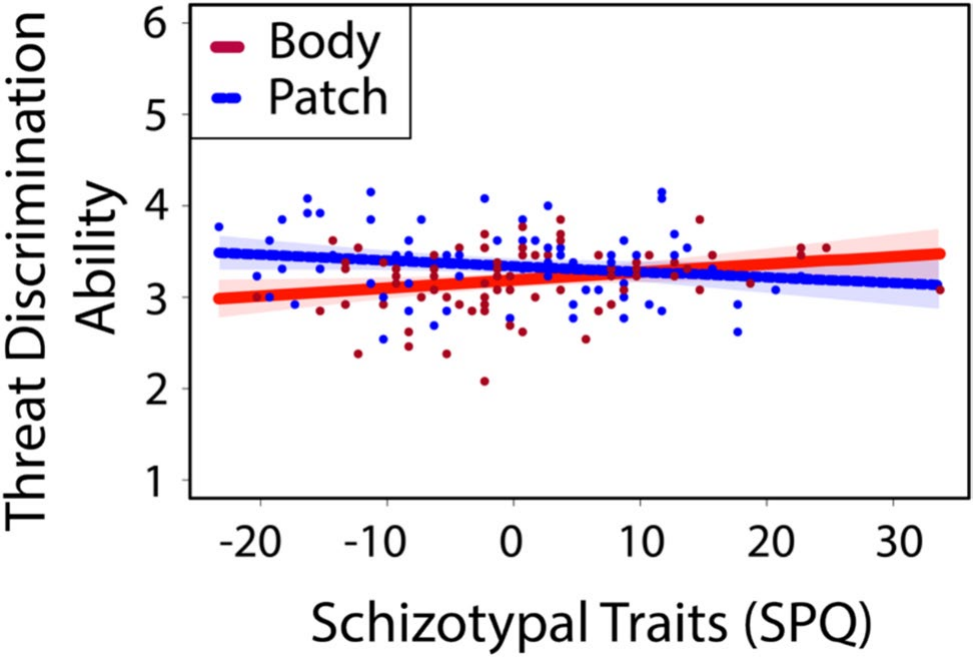
Simple slopes between mean-centered schizotypal traits (composite measure) and threat discrimination vary across different display types. With reduced social context (Body Display; red), high schizotypal traits show better threat detection ability (*threat difference* = 3.184 +.009 × SPQ). With increased social context (Patch Display; blue dashed line), high schizotypal traits showed a reduction in threat discrimination ability (*threat difference* = 3.342 −.006 × SPQ), *p* =.007. Data points represent individual observations, color coded by display type. Red and blue shaded areas represent 95% confidence intervals for each display type. (Color figure online)

To account for collinearity, we first computed correlations between individual difference measures. AQ and SPQ scores were highly correlated, spearman’s $\rho$ =.624, *p* <.001 as well as between many of the subtypes (reported in Fig. 5). Given the high multicollinearity, we conducted separate moderation analyses for each individual difference measure. For composite SPQ traits, the model was significant with the interaction term, Δ*R*^2^ = 4.42%, Δ*F*(1,153) = 7.407, *p* =.007 (Fig. 6). The direction of the interaction was revealed by regression equations for each display. For the body display, *threat difference* = 3.184 +.009 × SPQ, indicated a positive relationship between threat discrimination and SPQ traits. In contrast, a negative relationship was found between threat discrimination and SPQ traits in patch display (*threat difference* = 3.342 −.006 × SPQ). The reverse directionality of the moderation between display types revealed that additional social context conveyed by the patch display negatively influenced threat discrimination in high schizotypal traits, while reduced social context in the body display improved threat discrimination in high schizotypal traits.

We parcellated the SPQ composite into its subtypes and examined their individual contributions. Three subtypes were significant using the same procedure as above: SPQ suspiciousness subtype (SPQ-Sus), Δ*R*^2^ = 6.70%, *F*(1,153) = 11.581, *p* <.001, with a direction reversal in the moderation effect between display types: patch display, *threat difference* = 3.336 −.054 × SPQ-Sus; body display, *threat difference* = 3.336 +.044 × SPQ-Sus; SPQ unusual perceptual subtype (SPQ-Unusual), Δ*R*^2^ = 3.30%, Δ*F*(1,153) = 5.508, *p* =.020: patch display, *threat difference* = 3.342 −.033 × SPQ-Unusual; body display, *threat difference* = 3.185 +.041 × SPQ-Unusual, and SPQ Odd Beliefs (SPQ-Beliefs), Δ*R*^2^ = 3.10%, Δ*F*(1,153) = 5.096, *p* =.025: patch display, *threat difference* = 3.346 −.060 × SPQ-Beliefs; body display, *threat difference* = 3.190 +.038 × SPQ-Beliefs.

Ratings on nonthreatening videos correlated with both SPQ-Beliefs ($\rho$ =.255, *p* =.025) and SPQ-Sus ($\rho$ =.264, *p* =.020) traits in the patch display, indicating that individuals with more suspiciousness and odd belief traits rated nonthreatening videos more threatening in the presence of more social context, thereby reducing their threat discrimination ability. However, when social context was reduced in the body display, ratings on nonthreatening videos negatively correlated with more SPQ-Unusual traits, $\rho$= −.268, *p* =.016, suggesting that individuals with more unusual perceptual experience traits better identified nonthreatening videos when they were primarily reduced to body movements. No other SPQ traits were moderated by display type (SPQ-Anxiety, *p* =.516; SPQ-Constricted, *p* =.132; SPQ-OddSpeech, *p* =.110; SPQ-NoFriends, *p* =.247; SPQ-Odd Behavior, *p* =.072; SPQ-Reference, *p* =.148).

**Fig. 7:**
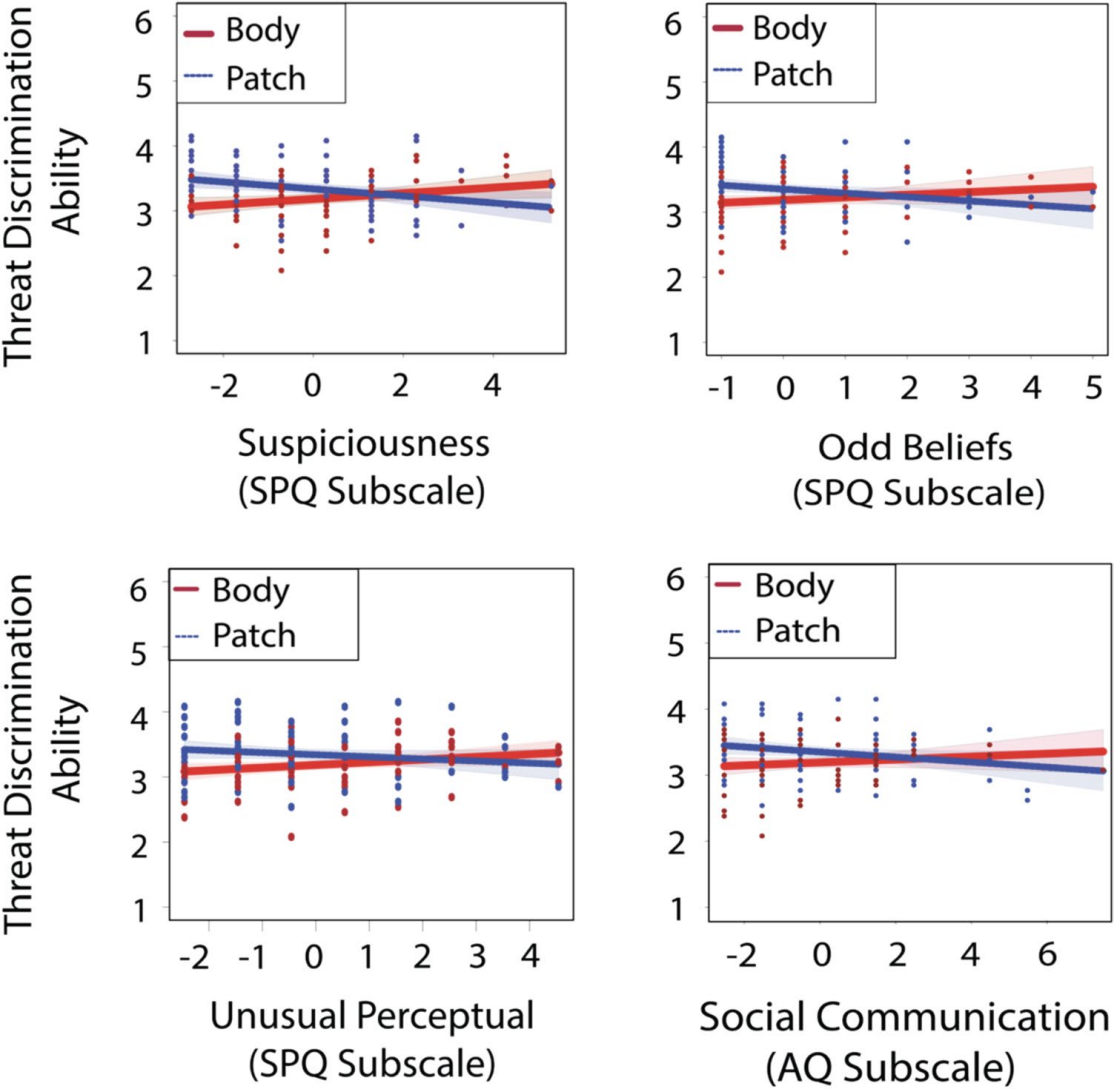
Simple slopes between mean-centered individual differences and threat discrimination ability (*y*-axes) as a function of display type. *Top:* schizotypal suspiciousness traits (*left panel);* schizotypal odd beliefs traits (*right panel*) *Bottom:* schizotypal unusual perceptual traits (*left panel);* autism social communication traits (*right panel*). With reduced social context (Body Display; red), higher traits on all scales showed better threat detection ability: SPQ suspiciousness, *threat difference* = 3.336 +.044 × SPQ suspiciousness; SPQ Unusual, *threat difference* = 3.185 +.041 × SPQ-Unusual*;* AQ Communication, *threat difference* = 3.198 +.025 × AQ communication; SPQ Odd Beliefs, *threat difference* = 3.190 +.038 × SPQ odd beliefs. With increased social context (Patch Display; blue dashed line), higher traits showed a reduction in threat discrimination ability: SPQ suspiciousness, *threat difference* = 3.336 −.054 × SPQ suspiciousness; SPQ Unusual: threat difference = 3.342 −.033 × SPQ-Unusual; AQ Communication: *threat difference* = 3.358 −.041 × AQ communication; SPQ Odd Beliefs, threat difference = 3.346 −.060 × SPQ Odd Beliefs. Data points represent individual observations, color coded by display type. Red and blue shaded areas represent 95% confidence intervals for each display type. (Color figure online)

No moderation effect was found for composite AQ scores (*p* =.089). However, a moderation effect was present for subscale AQ traits linked to social communication (AQ-Comm), Δ* R*^2^ = 3.00%, Δ* F*(1,156) = 5.140, *p* =.025; patch display, *threat difference* = 3.358 −.041 × AQ-Comm, body display, *threat difference* = 3.198 +.025 × AQ-Comm. Follow-up correlations revealed that individuals with more autism-spectrum social communication traits accurately discerned threatening videos as more threatening when the interactions were primarily reduced to body movements in the body display ($\rho$=.246, *p* =.028) (Fig. 7). No other AQ subscales were moderated by display type (AQ-Attention: *p* =.531; AQ-Detail: *p* =.093, AQ-Social: *p* =.200, AQ-Imagine: *p* =.093).

## Discussion

In a large sample of participants, we found both an impairment and advantage in threat detection based on trait variance, solely by manipulating the degree of social context in the stimulus. Specifically, high schizotypal trait individuals and subprofiles of schizotypy and autism showed poorer threat discrimination in the presence of more social context. However, when social context was reduced, these individuals instead better discriminated threat relative to low-trait individuals.

Why might social context impair threat detection in high schizotypal trait individuals? Individuals on the schizophrenia spectrum are hypersensitive to social stressors in the environment (Veling et al., 2016), measured by gaze avoidance at social scenes (Freeman et al., 2000; Green & Phillips, 2004; Phillips et al., 2000; Li et al., 2020), aberrant processing of socio-emotional faces (Nikolaides et al., 2016), increased paranoia in the presence of more social context (Veling et al., 2016), and overall difficulties in social cognition (M. F. Green et al., 2015). Profiles of schizophrenia also experience dysfunctional sensory modulation, including sensory overresponsivity (Sanchis-Asensi et al., 2023; Schwarzlose et al., 2023), and more attunement to visually distracting social information in the environment. Abnormal sensory processing is further considered a marker of psychosis, often emerging prior to its onset (Lipskaya-Velikovsky et al., 2015). Such experiences can be debilitating and can increase self-reported anxiety while also reducing perceived social support (Kinnealey et al., 2011).

To examine why social context impaired dynamic threat discrimination in our nonclinical sample, we turned to examine the subscale variance. Examining subscale variance in a nonclinical cohort can shed light on the proneness toward developing schizophrenia. Further, in using a nonclinical population, we can examine liable trait variance for developing schizotypy.

We found that social context specifically affected individuals with high suspiciousness tendencies, odd beliefs, and unusual perceptual experiences. Suspiciousness on the SPQ measures the tendency toward paranoia and delusions and is a highly common psychotic experience that emerges prior to first-onset psychosis (An et al., 2010). The odd beliefs subscale of the SPQ measures magical thinking, such as conspiracy ideation and supernatural beliefs (Barron et al., 2014; Darwin et al., 2011; Raine, 1991). The unusual perceptual subscale measures proneness to sensory distortions, such as hallucinations and magical thinking to an extent (Raine, 1991). Together, these subscales (suspiciousness, odd beliefs, and unusual perceptual experiences) all fall under the cognitive-perceptual, or positive symptomatology of schizotypy, and are associated with anomalous salience detection, such as pattern detection in noise, and a tendency to find meaning in ambiguous input (Corlett et al., 2010; Garety et al., 2001), such as socially neutral contexts. In our data, individuals high on each of these traits rated nonthreatening videos as more threatening in the presence of more social context, which correspondingly reduced their ability to discern threat between the threatening and nonthreatening interactions. It is possible that individuals who experience more of these traits may be unduly influenced by social information and overmentalize (Okruszek et al., 2015)— ascribing greater threat to nonthreatening situations (Veling et al., 2016).

In recent work, higher levels of paranoia, as well as ideation in conspiracy beliefs, increase threat perception in neutral contexts (Greenburgh & Raihani, 2022). Individuals with persecutory delusions experience abnormal perceptions of threat, even in socially ambiguous contexts (Freeman et al., 2000; Phillips et al., 2000). We found that after reducing social context in the video, threat detection was not only preserved, but individuals with more of these traits showed a superiority effect, with better threat discrimination when the interactions were primarily reduced to body movements. For the first time to our knowledge, this revealed both a threat detection impairment and advantage in high schizotypal trait individuals, observed between different social context conditions. This suggests that the ability to distinguish threat based on differentiating degrees of social context may hold potential as a marker for schizotypal proneness or prognosis. 

Since both the schizophrenia spectrum and autism spectrum share etiological and phenotypic overlap (Couture et al., 2010; cf. Hudson et al., 2023) and threat detection has also been shown to be affected in the autism spectrum (e.g., Krysko & Rutherford, 2009), we further examined the relationship between threat detection and autistic traits. While AQ composite scores were nonsignificant on threat discrimination, AQ social communication moderated threat discrimination between display types. AQ social communication overlaps in its etiology with negative symptoms in schizophrenia (Spek & Wouters, 2010; Tordjman, 2008; cf. Hudson et al., 2023) and is linked to atypical processing of biological motion (Wang et al., 2018), and communicative difficulties (Baron-Cohen et al., 2001). The superior performance shown in these individuals when social context was reduced could be attributed to a similar compensatory mechanism in biological motion to help accurately perceive the social scene. However, since these conditions are not unidimensional, more research using parcellations of the subscale variance is needed to shed light on the exact underlying mechanisms for a possible superiority effect. Moreover, since our findings reflect trait-level variance in the general population, caution must be taken when drawing conclusions to the larger clinical population.

In summary, we found that accurate threat discrimination depends on the degree of social context in schizophrenia and autism-spectrum subtypes. These findings hold numerous implications for targeted treatment interventions in these conditions and the potential to use deep learning for realistic manipulations of social environments. Rather than domain-general impairments in threat processing, dysregulated social perception may be especially pronounced in these conditions, which in turn could affect threat detection ability. This may be driven by “seeing threat when not there,” as found in the schizophrenia trait parcellations. Interventions that simplify social information, such as removing facial cues, may offer a practical way to improve threat detection and support well-being of individuals in these conditions.

## Data Availability

Data, materials, and code for the experiment and analyses is made publicly available on OSF (https://osf.io/thgan).
